# Coverage Effects in Quartz Crystal Microbalance Measurements with Suspended and Adsorbed Nanoparticles

**DOI:** 10.1021/acs.langmuir.3c02792

**Published:** 2023-12-21

**Authors:** Rafael Delgado-Buscalioni

**Affiliations:** Departamento de Física de la Materia Condensada, Universidad Autonoma de Madrid, and Institute for Condensed Matter Physics, IFIMAC. Campus de Cantoblanco, Madrid 28049, Spain

## Abstract

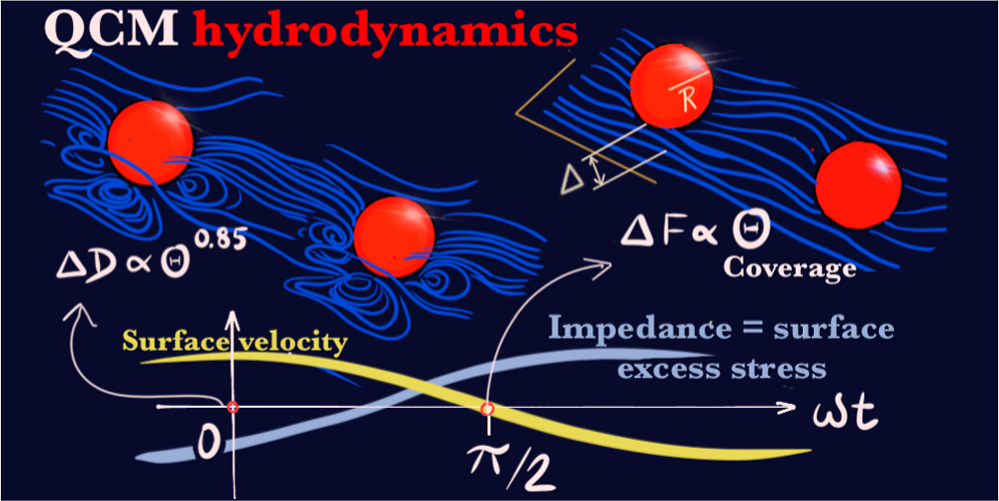

Quartz crystal microbalance (QCM) biosensors often deal with nanoparticles suspended in the solvent at tens of nanometers above the resonator while being linked to some molecular receptor (DNA, antibody, etc.). This work presents a numerical analysis based on the immersed boundary method for the flow and QCM impedance created by an ensemble of spherical particles of radius *R* at varying surface coverage Θ and particle-surface gap distance Δ. The trends for the frequency Δ*f* and dissipation Δ*D* shifts against Θ and Δ are shown to be determined by modifications in the structure of the perturbative flow created by the analytes. Simulations are in good agreement with a relatively large experimental database collected from the literature. Qualitative differences between the adsorbed (Δ ≈ 0) and suspended states (Δ > 0) are highlighted. In the case of adsorbed particles, deviations from the linear scaling Δ*f* ∝ Θ are observed above Θ > 0.05 and largely depend on the specific analyte–substrate combination. Moreover, in general, Δ*D*(Θ) is not monotonous and usually presents a maximum around Θ ∼ 0.2. In the case of suspended analytes, the agreement with the numerical results is quantitative, indicating that the predicted scalings are universal and determined by hydrodynamics. Up to high coverage, the suspended particles present Δ*f* ∼ Θ and Δ*D* ∼ Θ^β^, where β ≈ 0.85 is not largely dependent on *R*. The present findings should help forecast molecular configurations from QCM signals and have implications on QCM analyses, e.g., in the case of suspended ligands (Δ*f* ∝ Θ), it is safe to use Δ*f* to build Langmuir isotherms and estimate equilibrium constants. Open questions on the transition from the suspended-to-adsorbed state are discussed.

## Introduction

The quartz crystal microbalance (QCM) is an essential technique for soft matter analyses, and is applied to supported lipid membranes, proteins, antibodies, DNA, liposomes, bioconjugated nanoparticles, biological cells, and all types of viscoelastic materials. QCM is label-free, relatively cheap, easy-to-use, broadly applicable, and extremely sensitive. These properties make QCM one of the workhorses for molecular sensing in biological and medical assays, nanotoxicity, water pollution, and more.^1−3^ Despite the tremendous technical advances in QCM since its original design in vacuum,^4^ in liquid environment, the QCM has not reached its full analytic potential due to the dominant role of the complex hydrodynamic interactions among the analytes and the oscillating surface. To further complicate things, biostructures are generally flexible (dissipative), and their stress present viscoelastic response. Despite these difficulties, the extreme sensitivity of QCM has so far offered a useful way to rationalize experimental observations by comparisons based on phenomenological descriptions, such as electrical circuits,^3^ couple-resonators with effective springs and dampers,^5,6^ or different types of trapped solvent models,^7,8^ which were introduced to rationalize the strong overestimation of the Sauerbrey relation (SR) on the mass adsorbed over the QCM surface, when used in liquids.^9^

Theoretical and numerical works are needed to gradually translate the QCM signals (frequency Δ*f* and dissipation Δ*D* shifts) into a more accurate representation of the physical configuration and mechanical properties of discrete analyte deposits. The importance of hydrodynamics in QCM was indeed recognized in pioneer numerical analyses,^10,11^ and it has been receiving increasing attention in recent years. The lattice Boltzmann method has been used to simulate rigidly adsorbed particles,^12,13^ while the immersed boundary (IB) method has been used to analyze suspended liposomes linked by DNA strands.^14^ This work extends the latter study to unveil the effect of increasing the surface coverage. Advances in other aspects of QCM (pumping flow, kinetics, viscoelasticity) are also ongoing.^2,15^

Recent theoretical works^9,16,17^ clearly demonstrate that in liquids, the main source of QCM impedance has a hydrodynamic origin. The disturbance flow created by the particles propagates the (hydrodynamic-induced) particle stress toward the surface, even when the particle is not in direct contact with the surface. In the linear regime [also called small-load approximation (SLA)] Δ*f* ≪ *f*, the QCM apparatus essentially senses the tangential stress at the oscillating surface, which is proportional to the impedance. If the particles are (strictly) suspended, the excess wall stress is uniquely created by the particle-induced hydrodynamic perturbation. But hydrodynamics is the dominant source of impedance even if the particles are tethered by molecular linkers^14^ or even if the particles are adsorbed.^9^ A similar conclusion has been reached in a recent theoretical work.^17^ In view of its dominant role in the QCM signals, understanding “QCM hydrodynamics” is the first step to unravel the often secondary, yet extremely conceptually relevant, questions on the molecular-specific effects arising from the physicochemical interplay of the sample, substrate, and solvent.

Theoretical analyses of the QCM flow created by an isolated particle (infinitely dilute regime Θ → 0) are starting to appear.^9,16−18^ Theoretically, “QCM hydrodynamics” is connected to the long-standing problem of flow disturbances in the semibounded unsteady Stokes flow.^19−22^ Analytical relations for the impedance of adsorbed particles were first proposed in ref (9) showing a good agreement between the numerical and experimental results, however due to a cancellation of errors. In fact, the recent theoretical analysis of Leshansky et al.^17^ corrected and expanded the analysis of ref (9) by including the effect of the particle perturbative flow. That work^17^ used an elegant numerical way to calculate the impedance in the infinitely diluted regime (Θ → 0) based on the Lorentz reciprocal theorem (LRT) and derived closed analytical expressions for the impedance, including the small-particle and long-distance limits.^17^ It shall be later shown that the results hereby presented for the impedance of suspended particles in the Θ → 0 limit agree with the results of ref (17). However, in the adsorbed state, the theory of ref (17) presents a strong discrepancy with the experimental trends and present numerical results for Δ ≈ 0. The origin of this discrepancy is briefly discussed by arguing that adding contact forces should conform the theory to the experimental trends. Yet, the details of the suspended-to-adsorbed transition where lubrication forces dominate are still not completely understood, and further work is needed.

The relation of Δ*f* and Δ*D* with the particle coverage Θ and the particle-surface gap distance Δ = *z*_p_ – *R* (here, *z*_p_ is the height of the particle center) are two extremely relevant pieces of information in QCM-applied domains. Theoretical predictions for Δ would be extremely useful in QCM studies of interfacial properties of charged colloids in electrolytes^6,23^ and notably, also in biosensors. Many QCM biosensors deploy molecular complexes involving linkers tethered to the surface, which specifically bind to the sampled molecule;^24,25^ or vice versa; they detect surface-tethered molecules by binding them to large particles (which enhance the QCM signals) such as the DNA–liposome complex tested in ref (26). Multilayers and multiple receptor–ligand steps are also frequently used in QCM biosensing, which might require optimizing Δ via the linker length.^27^ The coverage dependence is an even longer-standing problem in QCM analyses. For example, knowledge of the Δ*f*(Θ) relation would provide more accurate measurements of molecular dissociation constants via adsorption isotherms or better analytical estimations for the limit of detection of analyte concentration in bulk. In this sense, the excellent agreement between numerical calculations presented hereby for varying coverage and experiments for liposome–DNA complexes^26^ permit the forecast of the liposome coverage Θ, which is not accessible in experiments.

The Results and Discussion section starts by presenting the experimental raw data used for validation along with the numerical method and impedance expressions. It follows with an analysis relating the changes in the perturbative flow with the modifications in the QCM signals (Δ*f* and Δ*D*). The effect on the impedance is considered first in the dilute limit Θ → 0, where a comparison is made with the theory by Leshansky et al.^17^ Results at finite coverage bring the main conclusions about Δ*f* and Δ*D* coverage scaling for the suspended and adsorbed cases, which are validated by comparison with experiments. A discussion on the implications of the present findings on the applied QCM field and on the suspended-to-adsorbed transition in relation with the theory of ref (17) is then presented followed by conclusions and open questions.

## Materials and Methods

### Experimental Database

The numerical results presented in this work will be compared with the experimental data collected from the literature both for suspended particles (tethered to the surface by a molecular linker) and for adsorbed particles. The analytes used in the experiments are very different in nature (liposomes, exosomes, viruses, proteins, silica nanoparticles, etc.) and correspond to roughly spherical quasi-neutrally buoyant particles whose density is close to that of the solvent.

#### Experiments with Suspended Analytes

This set of experimental data includes results from two different studies on liposome–DNA complexes^26^ and on tethered exosomes.^25^ Both databases include results at low concentration, which are particularly useful to unveil scaling details. Data for the liposome–DNA complexes is publicly available in the CATCH-U-DNA project repository.^28^ The liposome–DNA complexes were formed by sequential injections to the QCM chamber of neutravidin (NAv), double-stranded DNA (dsDNA), and finally liposomes.^14^ Experiments were performed in water, using short double-stranded DNA strands with 21, 50, and 157 base pairs (bp), corresponding to DNA contour lengths of 7, 17, and 53 nm, respectively. Different set of complexes were formed using 1-palmitoyl-2-oleoyl-sn-glycero-3-phosphocholine (POPC) liposomes of radius *R* = 15, 25, 50, and 100 nm. DNA strands are attached on one side to the surface-adsorbed NAv through a biotin linker, while the other end of the DNA strand bears a cholesterol molecule, which anchors to the POPC due to its strong affinity (see ref (26) for details).

Experiments reported by Guldin et al.^25^ used exosomes with an average size of about 93 nm diameter. These extracellular vesicles are heterogeneous biomolecular structures enclosed by a lipid layer, with larger polydispersity in size, compared with that of the extruded liposomes of the previous data set. The exosomes were linked to the surface using a biomolecular complex formed by a biotinated antibody cleaved to the membrane, linked to its opposite side to a streptavidin which also links to a biotinated-PEG oligomer, which is tethered to the surface (see ref (25) for details). Exosomes were spiked both in human serum and in pure HBS solvent.

#### Experimental Data for Adsorbed Particles

This set of data was obtained from the relatively more abundant literature (part of this database was collected in ref (9)) and contains adsorbed proteins, viruses, and supported unilamellar vesicles (SUVs) reported by Bingen et al.;^7^ the first layer of adsorbed streptavidin reported by Steinmetz et al.,^29^ adsorbed polymer nanoparticles by Adamczyk and Sadowska,^8^ the results for liposomes at temperature *T* = 10 and 32*°* by Reviakine et al.^30^ (estimated heights of 91 and 65 nm), data for cowpea mosaic virus (CPMV) by Tellechea et al.,^10,11^ and latex nanoparticles on silica or on alumina-coated surfaces by Olsson et al.^5,6^ Additional data using high-frequency (150 MHz) QCM sensors^31^ from AWSensors were taken from the CATCH-U-DNA public repository:^28^ in particular, *R* = 50 nm liposomes tethered to short DNA chains (21bp) and NAv proteins.

### IB Method for QCM Hydrodynamics

This section briefly introduces the fundamentals of the IB method applied to a QCM flow. Details on the IB method can be found in ref (32)–^35^ and in ref (14) for the application of IB to a QCM flow. QCM sensors are based on the inverse piezoelectric effect, which by imposing an AC potential to a cut of crystal quartz induces shear waves at the material whose surface oscillates at MHz frequency. Computationally, the oscillation of the resonator surface is located at *z* = 0 and moves in the *x* direction with velocity *v*_wall_ cos(ω*t*). The flow velocity is assumed to satisfy the no-slip boundary condition **v** = 0 at *z* = 0 and vanish far from the resonator (**v** = 0 for *z* → 0). In the absence of analytes, this flow corresponds to the Stokes flow, which can be written as **v**_0_(*z*, *t*) = Re[*v*_wall_*e*^–α*z*^*e*^–iω*t*^] × **x**, with α = (1 – i)/δ. The penetration length δ = [2η/(ρω)]^1/2^ [of order *O*(100 nm) in water] roughly corresponds to the layer of fluid set in motion, being determined by the balance between shear flow advection and vorticity diffusion. The analyte creates a perturbation to the base Stokes flow, which is responsible for most of the excess stress at the surface. This excess stress is detected upon frequency and dissipation shifts over the bare flow levels. The total flow can be written as , where the perturbative flow velocity field is **ṽ**. Following the induced force formalism,^36^ the fluid momentum equation reads
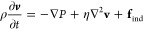
1with no-slip at *z* = 0 [i.e., **v**(*z* = 0, *t*) = *v*_wall_cos(ω*t*) **x**] and zero velocity at *z* → *∞*. The induced force density **f**_ind_(**r**) created by the particle guarantees that the fluid velocity equals the particle velocity at each position of the particle.^20,36^ The IB method^32,34,37,38^ solves the fluid phase using a regular mesh (cell side *h*) and introduces **f**_ind_(**r**) by a set of marker points along the immersed structure, generally connected with springs,^39^ which communicate with the fluid phase via distribution functions *S*(**r**) centered at each marker position **q**_*i*_ (here, three-point Peskin kernels^38^). In this way, **f**_ind_(**r**) = ∑_*i*_*S*(**r** – **q**_*i*_)**λ**_*i*_, where **λ**_*i*_ is the fluid-induced force to the particle marker *i*. The marker velocity  is coupled to the fluid velocity by the kinematic condition **u**_*i*_ = ∫**v**(**r**)*S*(**r** – **q**_*i*_)d*r*^3^ and momentum conservation ensured by . The excess mass  is the difference between the marker mass *m*_*i*_ and the fluid-displaced mass (the marker volume is ), and the force **F**_*i*_ collects internal (elastic) and any additional force (e.g., contact and or dispersion forces with the subtract). In this way, the IB method naturally resolves elastic structures in the flow, allowing particles to move freely according to the fluid-induced forces, elastic forces, and any other type of force.

The present IB calculations were performed using efficient codes running on graphical processor units, which have been adapted to the QCM setup. A set of calculations was done using the code FLUAM,^32−34^ now integrated in the code UAMMD(35) used to explore part of the system parameters in this work. Most calculations were carried out using the compressible scheme^32,40^ with a linear pressure–density relation, *P*(ρ) = *c*^2^ρ + *P*_0_. The sound velocity *c* was set large enough (ω*R*/*c* ∼ 10^–2^) to neglect the contributions from density waves over the oscillation frequency.^41^ More details on the computational scheme for the fluid solver can be found in.^32,34,40^

The particles used in this study are spherical and formed by an ensemble of nodes placed in an approximately hexagonal lattice and connected to their neighbors by strong springs, corresponding to the limit of a rigid particle. Typically, the neighboring nodes are placed at distances between 0.8 and 1.2 h. For comparison with experiments,^14,26^ some calculations include a semiflexible chain of beads (DNA strand) bonded to the spherical domain (liposome) and to the wall *z* = 0. The DNA strand is modeled as a semiflexible chain by connecting DNA markers by (3 body) angular springs with a persistence length of 50 nm. More details on the liposome–DNA model can be found in.^14^

### Impedance

The SLA (Δ*f* ≪ *f*) is usually fulfilled in experiments and guarantees that the flow is characterized by a single frequency so that any physical quantity can be described by a complex phasor. For instance, the fluid stress components σ_αβ_ = −*p* δ_αβ_ + η[∂_β_*v*_α_ + ∂_α_*v*_β_] can be expressed as
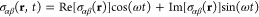
2

In the right-hand-side of the equation, σ_αβ_(**r**) is the phasor field [to avoid excessive notation, the same symbol has been used for the real-time field σ_αβ_(**r**, *t*)]. In the SLA, frequency and dissipation shifts Δ*f* and Δ*D* are proportional to the shifts in the impedance. In phasor terms, the total impedance *Z*_tot_ is the ratio of the total wall tangential stress Σ_tot_ ≡ ∫_*z*=0_σ_*xz*_d*x*d*y* and the resonator velocity, *Z*_tot_ = Σ_tot_/*v*_wall_. In the absence of analytes, the base flow impedance *Z*_0_ is easily obtained from its velocity phasor **v**_0_(*z*) = *v*_wall_exp(−α*z*)**x**, so that σ_0_ = (η∂_*z*_**v**_0_)_*z*=0_ and *Z*_0_ = −ηα. The total impedance is then *Z*_tot_ = *Z*_0_ + *Z*, where the *excess* impedance is noted as  (for notation simplicity, the tilde is removed from the excess impedance). In the SLA, the frequency and dissipation shifts are proportional to the excess impedance^3^
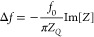
3
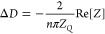
4where *f*_0_ = 5 MHz is the fundamental frequency of the quartz crystal cut and *n* is the overtone (*f* = *nf*_0_). *Z*_Q_ = 8.8 × 10^6^ kg/(m^2^ s) is the impedance of the quartz crystal cut.

The excess impedance *Z* created by the analytes has a contribution from the particle–substrate contact forces *Z*_c_ and a hydrodynamic part, the perturbative flow *Z*_h_ from the perturbative tangential stress at *z* = 0, i.e.,
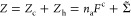
5where *n*_a_ would be the number of contacts per unit area and *F*^c^ is the contact force in *x*-direction averaged over contacts. The hydrodynamic stress is due to the perturbative flow, , where . Computationally, time-dependent fields are Fourier transformed using about 10–20 cycles to obtain the phasor fields (the first 5 cycles are excluded in this calculation to avoid the transient regime). Then, the base flow stress is subtracted to obtain the excess impedance and the frequency and dissipation shifts from eqs 3 and 4.

## Results and Discussion

### Perturbative Flow

**Figure 1:**
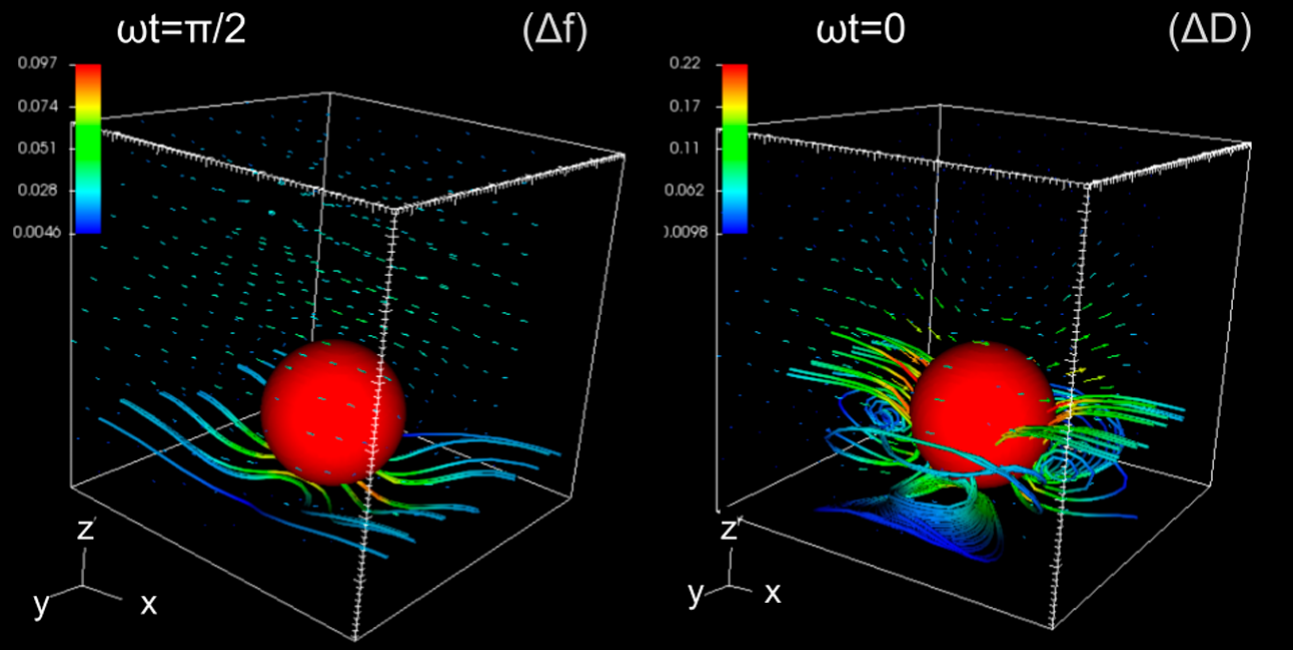
Streamlines of the perturbative flow near the resonator surface (*z* = 0) at ω*t* = π/2 and ω*t* = 0. The particle has a radius of *R* = 50 nm, and the gap distance is about Δ = 13.5 nm. The surface oscillates with small amplitude (about 4 nm) and a frequency of 35 MHz (fluid penetration length δ = 95 nm). The color code indicates the modulus of the disturbance velocity in units of the maximum resonator velocity (values in the legends). The setup corresponds to a periodic array of particles with a coverage of Θ = π*R*^2^/*L*^2^ = 0.12. Indications between brackets denote that the overall wall shear stress at ω*t* = π/2 provides Δ*f*, while at ω*t* = 0, it yields Δ*D* (see eqs 2–4).

A brief discussion of the 3D-perturbative-flow-created free suspended particles is now presented to shed information about the origins of the impedance scaling, as discussed in the next section. Figure 1 illustrates the 3D structure of the disturbance flow created by a rigid spherical particle of 50 nm radius placed at a distance of Δ ≈ 13 *nm* (surface-to-surface) submitted to a frequency *f* = 35 MHz in water (δ ≈ 95 nm). Calculations were performed in a tall box periodic in *x* and *y* directions (*L* ≡ *L*_*x*_ = *L*_*y*_). No-slip boundary conditions were imposed at the two extremes of the box *z* = 0 and *z* = *L*_*z*_ with **v**(*z* = 0) = *v*_wall_ cos(ω*t*)**x** and **v**(*z* = *L*_*z*_) = 0. The dimension of the box in *z* direction was chosen to be large enough to avoid spurious perturbations from the upper boundary condition: *L*_*z*_ ≥ 5 *L* was enough to avoid finite box effects.

The flow of Figure 1 corresponds to a single particle in the doubly periodic box (*xy* plane), thus representing a periodic array of particles with coverage Θ = π*R*^2^/*L*^2^, where *R* is the radius of the particle. The figure focuses on the streamlines nearby the wall region, and it shows two relevant instants of the cycle: zero phase ω*t* = 0 and at quarter-of-cycle ω*t* = π/2. These instants are particularly relevant because (see eqs 2–4) they connect the flow with the experimental QCM signals. In particular, at ω*t* = 0, the stress field is equal to the real part of its phasor; thus, from eq 4, its average at *z* = 0 is proportional to Δ*D*. In turn, at ω*t* = π/2, the imaginary part of the averaged wall stress provides the frequency shift Δ*f*.

**Figure 2:**
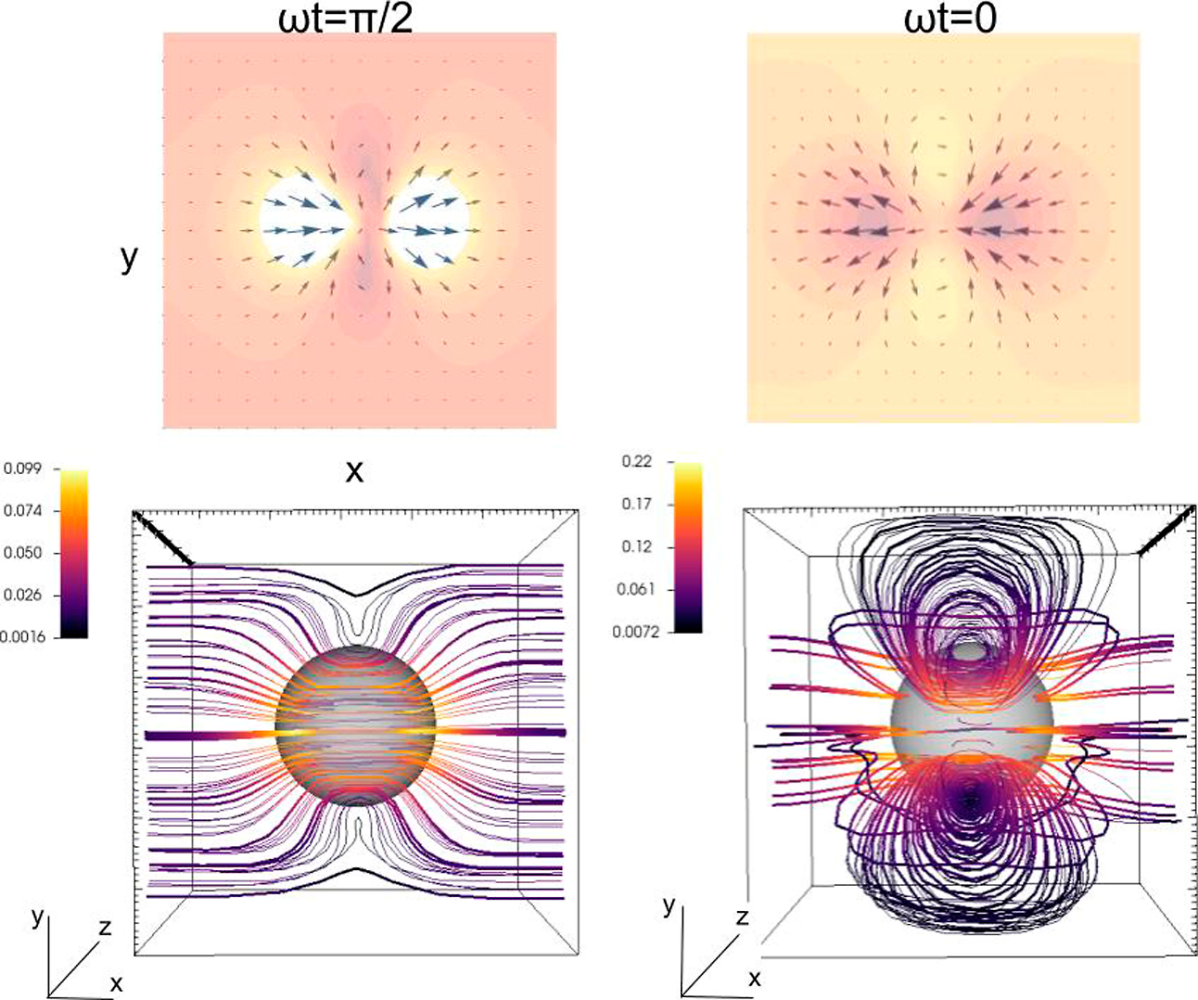
Detail of the perturbative flow of Figure 1 close to the wall. (top) Velocity field and colored contour plots for the wall shear stress. Bottom view of streamlines showing the formation of large lateral vortices over the *xy* plane at ω*t* = 0 (see also Figure 1).

At zero phase ω*t* = 0, the resonator velocity *v*_wall_ cos(ω*t*) reaches its maximum, and it becomes zero at ω*t* = π/2 (maximum deformation). At a qualitative level, this simple fact is the reason behind the stronger and more complex perturbative flow observed in Figure 1 at ω*t* = 0, shown by the streamlines nearby the surface. The magnitude of the perturbative velocity field |**ṽ**| (color-coded isovalues) shows that the perturbative flow is faster at ω*t* = 0 than at π/2 (typically, a factor two for the largest velocities). Also, the perturbative flow close to the surface has a more laminar structure at ω*t* = π/2 compared with the more complex vortical structures appearing at zero phase. One should expect that the disparity of the perturbative flow over the cycle (ω*t* = 0 and π/2) should be reflected in different scalings for Δ*f* and Δ*D*. Yet, the sole quantity sensed by the QCM is the averaged wall stress, so it is necessary to consider how the 3D flow projects on the *z* = 0 plane. In particular, the dissipation shift is generally positive Δ*D* > 0 (as in the case of Figure 1), meaning (see eq 4) that the average of  has to be negative. In turn, Δ*f* < 0, so the spatial average of  should be positive. Note that for large particles, a crossover to Δ*f* > 0 takes place^16^ (for *R*/δ > 3 in the case of adsorbed particles), yet this case is not analyzed here. Figure 2 shows the velocity field very close to the surface **ṽ**(*x*,*y*,*z* = *h*) (*h* is the fluid mesh) and the contours of the tangential wall stress. Note that the structure of the wall shear stress is similar to that in the results presented in ref (17).

Inspection of Figures 1 and 2 allows connecting the wall stress with the flow structure above. At ω*t* = π/2, the perturbative current which creates most of the wall stress is located below the particle and flows in the *x* direction, so that the shear rate is positive (contributing to Δ*f* < 0) almost everywhere. The situation is more complex at ω*t* = 0. Two corotating vortices circulating in the *xz* plane are formed in the fore-and-aft of the particle, precisely where the particle “pushes” the fluid leading to the largest pressure differences. At the surface *z* = 0, these vortices create domains with a negative shear rate, contributing to an increase in Δ*D*. But, furthermore, a pair of counter-rotating vortices appear at each side of the particle, unfolding over the *xy* plane (somewhat tilted with respect to the *z* direction). At *z* = 0, this lateral vortex pair creates positive shear, which contributes to a decrease in Δ*D*. As the particle-surface gap Δ is increased, this counter-rotating vortex pair becomes closer and spreads below the particle, setting the two negative-shear domains apart (as in Figure 1, see also the streamlines in the bottom view of Figure 2). As discussed later, this feature changes as Δ is decreased leading to relevant effects.

**Figure 3:**
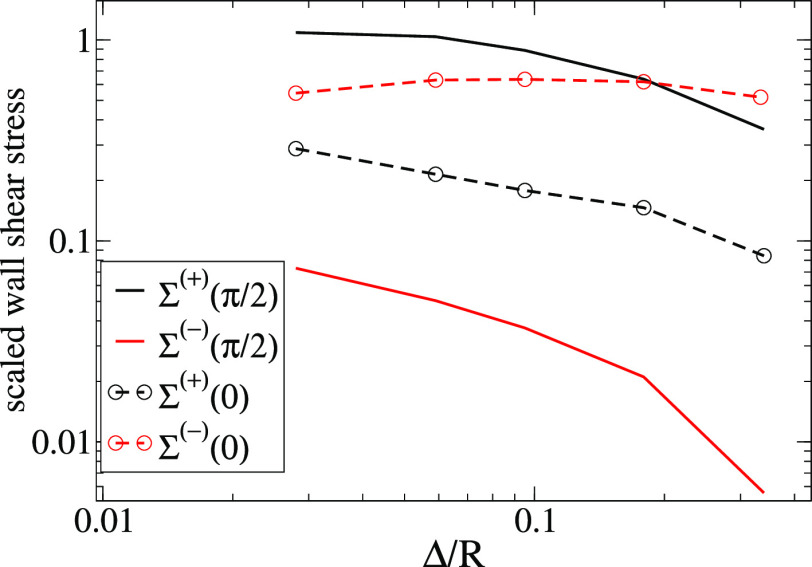
Positive and negative contributions to the total wall stress defined in eq 6 against the scaled gap distance Δ/*R*. Calculations correspond to *R*/δ = 0.53.

To rationalize the relevance of these flow structures on the QCM impedance, it is interesting to separate the total contributions from the domains with positive and negative stress. In particular,  with  if  and zero otherwise. The total perturbative stress is decomposed as
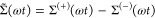
6(note that Σ^(−)^ is built to be positive). Figure 3 shows the variations of Σ^(+)^ and Σ^(−)^ with the particle-surface gap Δ for *R*/δ = 0.53 (same value as in Figure 1). As already revealed by inspection of the stress field, at ω*t* = π/2, the positive-stress domains are clearly dominant. For instance, for a *R*/δ ≈ 0.5 particle suspended at Δ/*R* ≈ 0.3, it is found that Σ^(+)^(π/2)/Σ^(−)^(π/2) ∼ 30, with this ratio being roughly independent of Θ. Besides, Σ^(+)^(π/2) increases roughly linearly with Θ (and so does |Δ*f*|, as shown later). Instead, at ω*t* = 0, the differences between Σ^(−)^ and Σ^(+)^ are smaller, leading to more complicated trends for Δ*D*. For the above example, Σ^(−)^(0)/Σ^(+)^(0) ∼ 4, but this ratio decreases as the particle approaches the surface (Figure 3).

**Figure 4:**
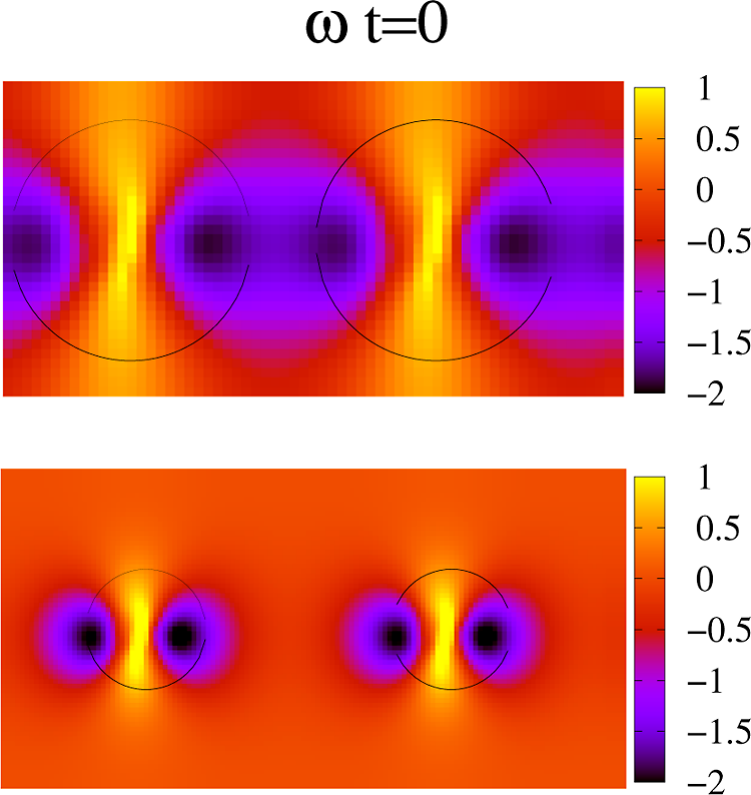
Contours of the tangential stress at the wall at ω*t* = 0 and Θ = 0.48 and 0.12 (bottom). Particle with *R* = 50 nm at Δ = 13.5 nm; penetration length δ ≈ 95 nm. The shear stress is scaled with η*v*_wall_δ.

**Figure 5:**
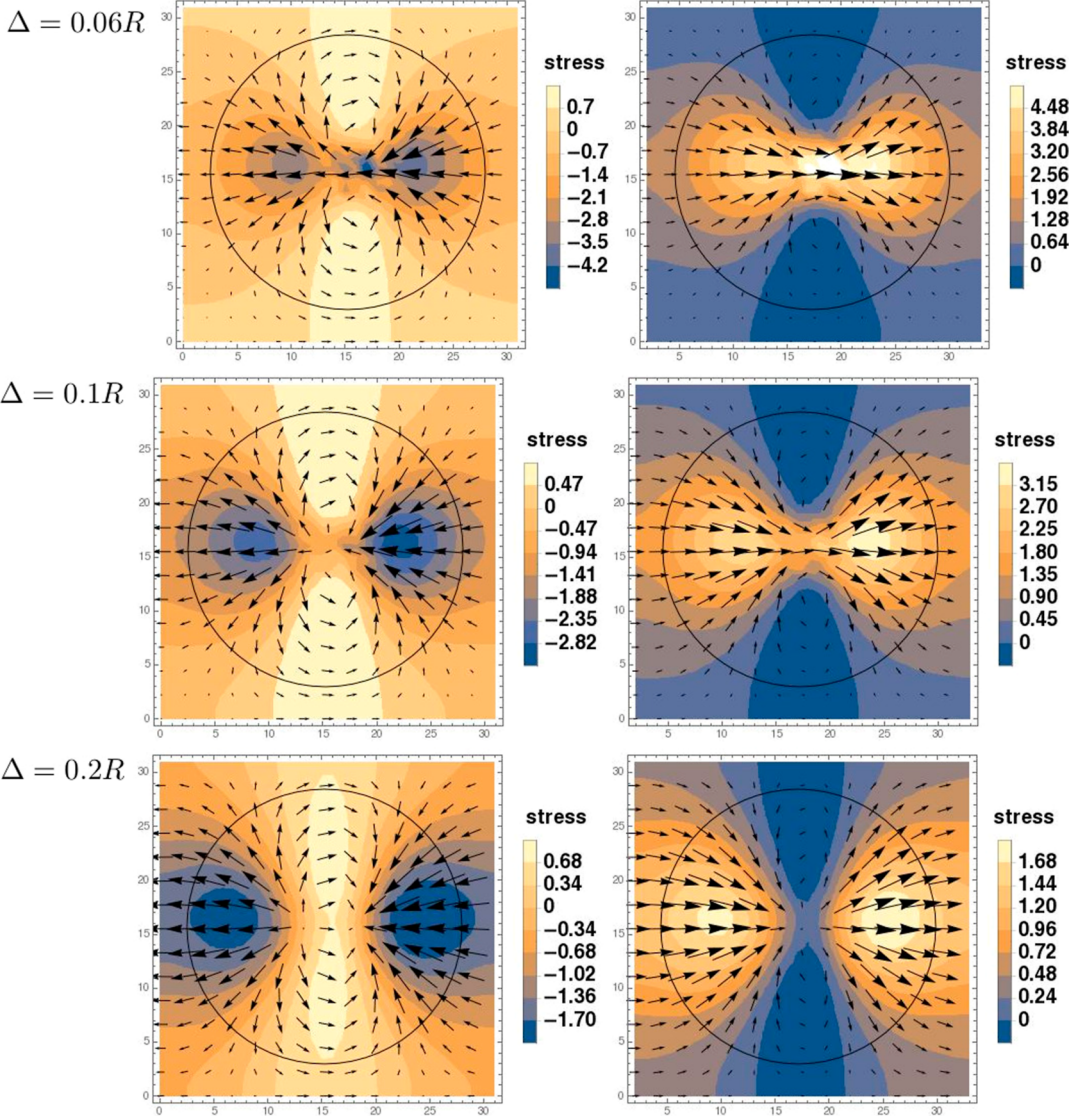
Perturbative flow in the *xy* plane close to the surface and tangential shear stress at the wall  for a particle of radius *R* = 50 nm and δ = 95 MHz. The streamlines are obtained from the projected vector field (*v*_*x*_, *v*_*y*_) at *z* ≈ 3 nm. From top to bottom, the scaled gap particle-surface distances Δ/*R* are approximately 0.06, 0.1, and 0.2. The net contribution of the positive and negative shear domains is shown in Figure 3. Left column corresponds to ω*t* = 0 and right column to ω*t* = π/2.

As the coverage is increased, the vortices created by the neighboring particles interact. The effect on the wall stress is illustrated in Figure 4. At high coverage, the qualitative differences in the flow structure alter the QCM signals. This path along the “suspended-to-adsorbed” transition is analyzed by comparing the stress domains (Figure 3) and the perturbative flow (Figure 5) close to the surface. All data correspond to Θ = 0.497 and *R*/δ ≈ 0.53. As the gap distance Δ is decreased, Figure 3 shows that Σ^(+)^(ω*t* = 0) gradually approaches the dominant negative wall stress Σ^(−)^(0), leading to a decrease in Δ*D*. The corresponding variation in the perturbative flow very close to the wall is illustrated in Figure 5 (colors indicate the scaled wall shear stress). As the particle approaches the surface starting from Δ = 0.2*R* in Figure 5, the fore-and-aft domains which contribute the most to the impedance merge below Δ/*R* ≈ 0.1 forming a single domain localized below the particle. Notably, at ω*t* = 0, this transition induces both a separation and an intensification of the counter-rotating lateral vortices, which strengthen the fluid-induced impulse^42^ and the (positive) wall stress, as seen by the inspection of the stress isocontours of Figure 5 and also in the net contribution Σ^(+)^(0) in Figure 3. As a consequence, below a certain gap Δ ∼ 0.1*R*, a fast decrease in Δ*D* is observed. This is confirmed by the impedance profiles presented below in Figure 7 (recall that −Re[*Z*] ∝ Δ*D*). The effect is stronger at high coverage, probably because the lateral vortices from the neighboring particles merge and reinforce each other. The situation at ω*t* = π/2 is qualitatively different because, even at large coverage and small Δ, the counter-rotating vortices in the *xy* plane are minor or even not developed (Figure 5). Consistently, the contribution of Σ^(−)^ remains marginal in Figure 3, which ultimately leads to a linear increase of Δ*f* with Θ in the case of suspended particles.

### Infinitely Dilute Limit

For completeness, this section presents the results for the impedance at infinitely small coverage Θ → 0. In a previous work^14^ on liposome–DNA complexes (suspended liposomes linked to DNA oligomers), numerical results using the present methodology were successfully compared with the experimental values obtained at low coverage (small |Δ*f*|). While the quantitative comparison with experimental values was quite satisfactory, the accuracy of such comparisons was limited by both the presence of the DNA-linker (whose effect starts to be significant for *R* < 25 nm^14^) and by the error bars intrinsic to any experiment. The recent theoretical study by Leshansky et al.^17^ allows for more finely tuned comparisons with the present IB method calculations. Leshansky et al. elegantly used the LRT to calculate the impedance (or equivalently the force acting on the surface). The theory starts by evaluating the impedance *Z*_s_ of a stationary particle over an oscillatory surface. Linear superposition leads to the impedance of a freely suspended particle *F* and to the impedance of an adsorbed particle *Z*_a_ which follows the surface as a whole, yet without exerting contact forces (or torque) with the substrate. Some aspects concerning the adsorbed state limit are discussed in the Conclusions section, where the main lines of the theory are depicted in more detail.

**Figure 6:**
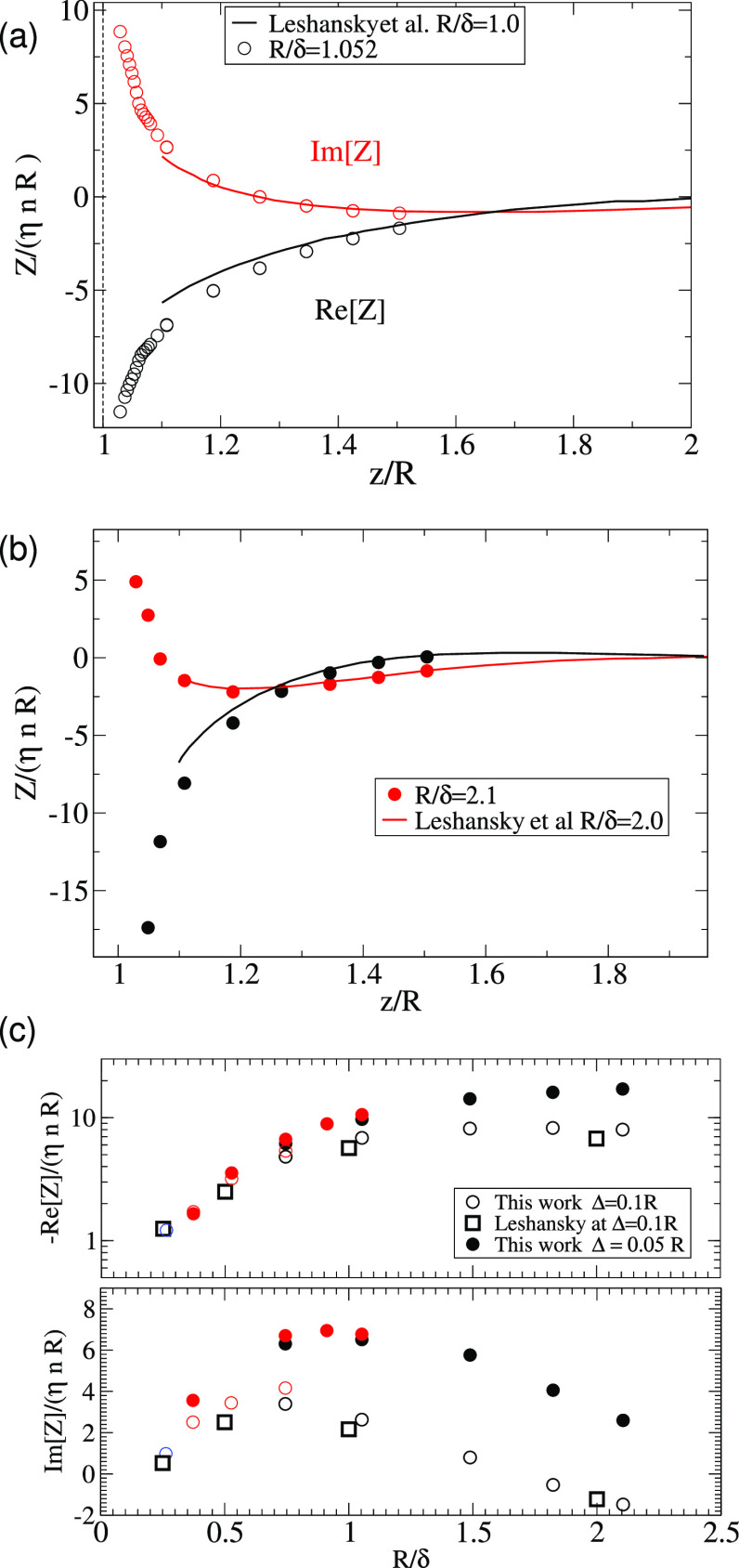
(a,b) Impedance against the height of the particle center over the resonator, scaled with its radius *z*/*R*. Present numerical results for (a) *R*/δ = 1.05 and (b) 2.1 (mesh size *h*/*R* = 0.04) are compared with finite element numerical evaluations in ref (17) for *R*/δ = 2 and 1.0. The nondimensional impedance *Z*/(η*nR*) equals the nondimensional force used in ref (17) (*n* is the particle surface density). (c) Comparison for the impedance evaluated at Δ/*R* = 0.1 against *R*/δ those presented in ref (17). Values of the mesh size used are *h*/*R* = 0.04 (black circles), 0.08 (red), and 0.12 (blue circle). Comparison with results for Δ/*R* = 0.05 indicates a significant increase in wall friction and impedance in large particles *R*/δ > 0.5, which becomes mild for smaller *R*/δ.

Numerical evaluation of the resistance functions required by the theory were evaluated in ref (17) using a finite element method with mesh size *h*/*R* = 0.05 within the domain. The resulting impedance profiles plotted against the scaled particle height (Δ/*R* + 1) were presented for Δ/*R* > 0.1 and properly merged, within the expected validity domains, with the analytical approximations derived for the small-particle and the long-distance limits.^17^Figure 6 (top) compares the profiles for the impedance versus particle height obtained from the present IB method calculations and the numerical evaluations by Leshansky et al. for free suspended particles. Figure 6 uses η*nR* as an impedance unit (where *n* is the particle surface density) so that the scaled impedance equals the scaled wall force used in ref (17) (*Z*/(η*nR*) = *F*/(η*Rv*_wall_)). The agreement is quite satisfactory. Note however that in Figure 6 (top), the particle radii chosen in both studies are slightly different. A more direct comparison in Figure 6 (bottom) shows the impedance evaluated at Δ/*R* ≃ 0.1 (the closest gap reported in ref (17)). Differences between the present calculations and those of ref (17) are less than about 5% for *h*/*R* < 0.05 and increase with the mesh-to-particle ratio *h*/*R*.

It is worth noting in Figure 6a,b that the sudden impedance increase observed as the particle approaches the surface is indicative of the lubrication regime. Close to the surface, numerical results for the particle velocity follow the scaling (e.g., *u*/*v*_wall_ – 1 = −*C*/| ln(Δ/*R*)| + *O*(1)) expected for the steady lubrication regime (note that vorticity diffusion along the small particle–resonator gap is much faster than the QCM period).^17^ However, a detailed analysis of the lubrication regime requires using a finer mesh size (notably for Δ < 0.05 *R*), and it is left for future work.

While in the case of suspended particles, the theoretical framework by Leshansky et al. finds excellent agreement with quite disparate numerical routes; the transition to the adsorbed state (Δ ≃ 0) poses a challenge. Both numerical results and experiments with adsorbates overwhelmingly present Δ*f* < 0 and Δ*D* > 0 (for *R*/δ < 3, see ref (16)). However, the adsorbate’s impedance predicted by Leshansky et al’s theory has the opposite sign (Im[*Z*_a_] < 0 and Re[*Z*_a_] > 0 corresponding to Δ*f* > 0 and Δ*D* < 0). The origin of this discrepancy is discussed in the Conclusions section.

### Impedance at Finite Coverage

**Figure 7:**
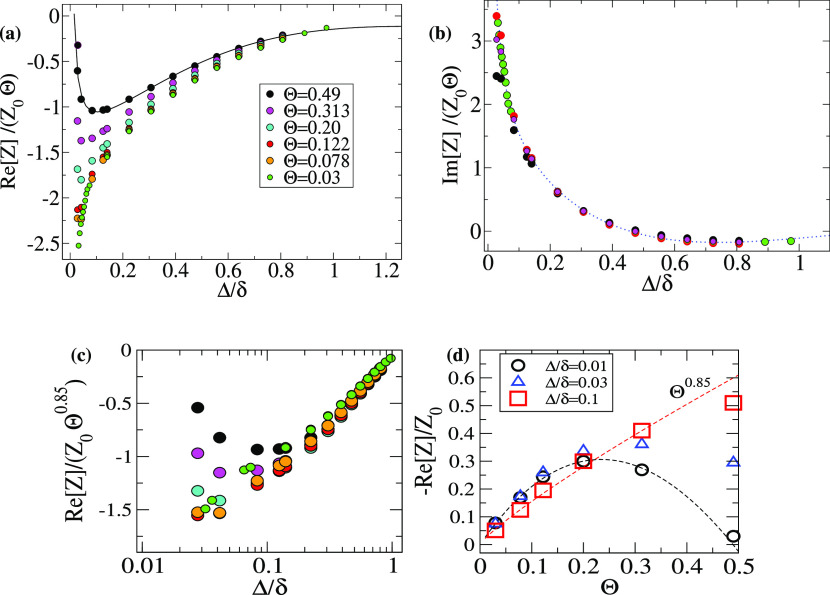
Real (a) and imaginary (b) part of the impedance *Z* scaled with *Z*_0_Θ (with *Z*_0_ ≡ η/δ) against the particle-surface gap distance Δ = *z*_p_ – *R*. Calculations correspond to a single particle of radius *R* = 50 nm in *xy* periodic boxes (equivalent to an array of particles with coverage Θ = π*R*^2^/*L*^2^). The penetration length is δ = 95 nm (35 MHz in water). (c) Re[*Z*] scaled with Θ^0.85^ showing a single trend for Δ/δ > 0.1 and (d) plotted against Θ at different heights (values for Δ = 0.01δ are extrapolations using fitting as the solid line in (a)).

This section presents the numerical results for the impedance at varying coverage and height starting with calculations using a single freely suspended spherical particle in a tall box periodic in the *xy* plane. This setup corresponds to a periodic array of particles with coverage Θ = π*R*^2^/*L*^2^. Figure 7 shows the results against the gap distance Δ = *z*_p_ – *R* at different coverages for particles with radius *R* = 50 nm and penetration length δ = 95 nm (35 MHz in water). It is noted that no contact (particle–substrate) forces were added in these simulations.

The impedance in Figure 7 presents qualitative differences as the gap is decreased or the coverage increased. First, scaling the impedance with the coverage *Z*/(*Z*_0_Θ) yields a master curve for the imaginary part of the impedance. Small deviations from this master curve are found at high coverage and are very close to the surface. From eq 3, this result predicts that |Δ*f*| ∝ Θ if the particles are suspended. As discussed above, this outcome is consistent with the excess wall stress being well localized in fore-and-aft regions around each particle at ω*t* = π/2. By contrast, the more complex flow structure at ω*t* = 0 leads to a more complicated dependence of Re[*Z*] with Θ and Δ. Figure 7c shows a sublinear increase Re[*Z*] ∼ Θ^0.85^ for any particle ensemble above Δ > 0.1δ (or Δ > 0.05*R*). This “power law” holds at least for Θ > 0.03. Below this small coverage, one may expect a linear scaling for Δ*D*(Θ), but the calculations were computationally impractical. As the particle approaches the surface (below Δ < 0.1δ), the coverage trends [Figure 7c] are strongly modified. Figure 7d shows that very close distance to the wall (nearly adsorbed configuration) |Re[*Z*]| has a nonmonotonous coverage dependence with a maximum value around Θ ∼ 0.2. By extrapolating the numerical data to Δ/δ = 0.01, one even predicts a crossover to “zero dissipation” (|Re[*Z*]| ∝ Δ*D* ≈ 0) for θ ≈ 0.5. Thus, the present analysis (for a periodic array of particles) predicts qualitatively different QCM responses for suspended particles (above Δ > 0.05*R*) and nearly adsorbed particles (i.e., at very small gap but without contact forces). As shown later, the experimental results for the real adsorbates present similar features as those of this nearly adsorbed case. In particular, the nonmonotonous trend for the “dissipation” Δ*D*(Θ), with a maximum value around Θ ∼ 0.2, is a well-known experimental feature for adsorbates.^10^ The analysis of the perturbative flow in the previous section clearly revealed its hydrodynamic, three-dimensional origin. Many important features of the QCM signals ultimately have a hydrodynamic origin, a conclusion already drawn in pioneer works.^7,10,11,30^ Comments on the implications of the present findings on standard QCM analysis protocols are given in the Conclusions section, and in particular, on the Δ → 0 extrapolation method for particle size determination.^3,7,11^

### Comparison with Experiments

#### Suspended Particles

**Figure 8:**
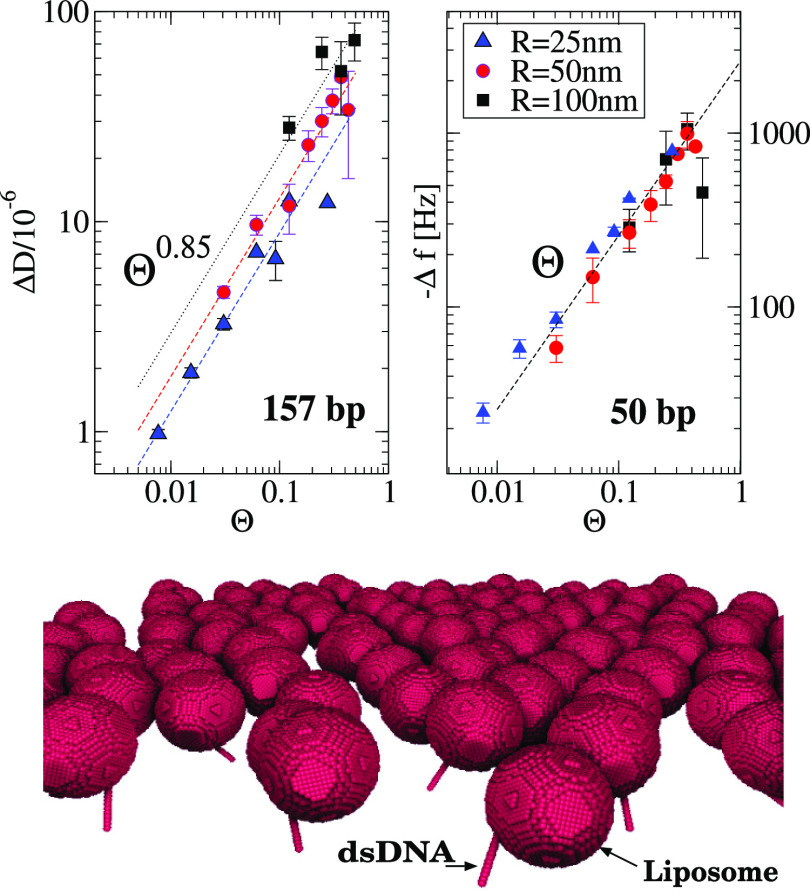
(Top) Values of Δ*D* and Δ*f* from simulations performed with *N*_L_ liposomes tethered to DNA strands at random locations and configuration over the surface. The horizontal axis is the particle coverage Θ = *N*_L_π*R*^2^/*L*^2^, where the resonator-plane side *L* ≈ 510 nm is fixed (the box is periodic in this plane). Dashed lines indicate the trends Δ*D* ∼ Θ^0.85^ and |Δ*f*| ∼ Θ. (Bottom) One of the configurations of the simulations, modeling 50 nm radius liposomes tethered to double-stranded DNA with 157 base pairs (157bp dsDNA).

In order to more closely reproduce the liposome–DNA experiments, a series of more computationally demanding simulations were performed in a tall box with a square (periodic) surface of *L* = 506 nm, containing a number *N*_L_ ∈ [1–20] of liposomes tethered to DNA strands. The setup is illustrated in Figure 8 (bottom). These liposome–DNA analytes were placed at random locations, with different chain-liposome configurations taken from the equilibrium distribution sampled by the Monte Carlo method. The coverage is now given by Θ = *N*_L_π*R*^2^/*L*^2^. About 10 runs with independent configurations were performed for each coverage. Deviations with respect to the results for the periodic array (single particle) taken at the average height of the multiparticle simulations were found to be within the standard deviation of the results for the many-body disordered state. It is also noted that for *R* > 25 nm, the contribution of the DNA strand was found to be negligible in comparison with the (hydrodynamic) contribution from the suspended liposomes.^14^

The outcome of these calculations, illustrated in Figure 8, presents some interesting features. In the case of Δ*f* and consistently with the result for the periodic array of particles in Figure 7, the disordered ensemble also presents a linear scaling for the frequency shift with coverage: |Δ*f*| ∝ Θ. Notably, if the particles are suspended, the linear trend remains up to high coverage Θ ∼ 0.5. It is advanced that this feature is not present in the adsorbed state. The other distinct feature of the suspended configuration is the sublinear scaling for the dissipation Δ*D* ∼ Θ^0.85^, which is also clearly reproduced in the disordered ensemble of particles (Figure 8). Thus, |Δ*f*| ∼ Θ and Δ*D* ∼ Θ^β^ with β ≈ 0.85 are the robust features of the QCM response of suspended particles. While the value of the exponent β was found to be roughly similar for particle sizes in the range *R* ≥ 25 nm (and δ = 95 nm) for smaller particles, it is possible that β presents slightly smaller values. For instance, simulation results for *R* = 15 nm present a slightly smaller value β ≈ 0.8; also, unpublished experimental results for tethered proteins confirm this trend. In conclusion, at the ω*t* = 0 phase, the perturbative flow structure induces long-ranged hydrodynamic interactions between particles, which persist up to small coverages. Below some threshold corresponding to ultralow coverage, one should expect to reach the infinitely dilute regime (for which Δ*D* ∝ Θ); however, it was not possible to reach this limit in the particle ensemble simulations as it requires extremely large boxes.

**Figure 9:**
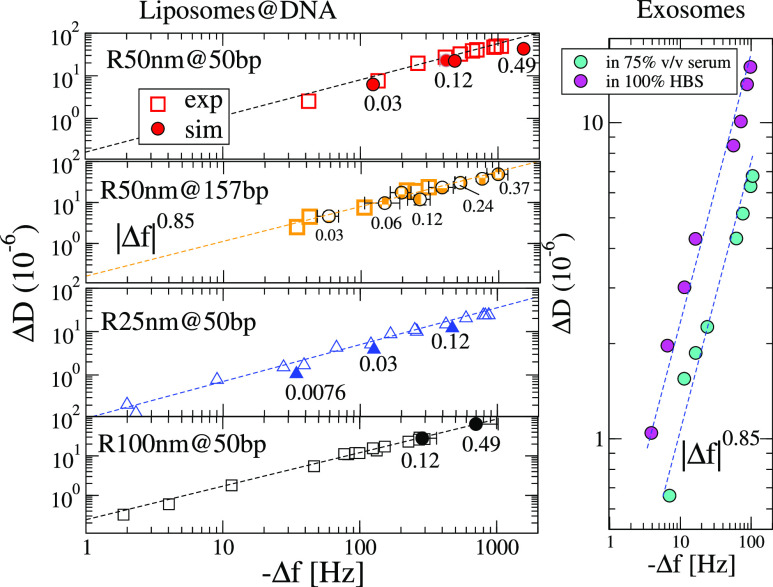
Dissipation Δ*D* versus frequency shifts Δ*f* for several types of suspended analytes. Left panels replot data from experiments with liposomes linked to DNA strands at low coverage in ref (26), being in quantitative agreement with the simulation results (filled symbols). The numbers beside the numerical results symbols indicate the particle coverage Θ. The notation *R* nm*@* bp indicates the liposome radius *R* (25, 50, and 100 nm) and the number of DNA base pairs (50 and 153 bp corresponding to contour lengths *L*_DNA_ = 17 and 53 nm) The right panel replots the data from experiments in ref (25) with exosomes suspended over macromolecular linkers and spiked in human serum and in pure HBS solvent. In the left panels (liposomes on DNA), the dashed lines correspond to Δ*D* = *a*|Δ*f*|^0.85^ with *a* = 0.10, 0.13, 0.16, 0.23, respectively, for (25 nm on 50 bp, 50 nm on 50 bp, 50 nm on 157 bp, and 100 nm on 50 bp) and those in the right panel to *a* = 0.15 and 0.33 for exosomes (average radius 46.5 nm) in human serum and HBS, respectively.

In order to validate the numerical predictions for suspended analytes, Figure 9 compares simulations with the experimental data for liposome–DNA complexes.^26^ An excellent quantitative agreement with numerical results is found for the different liposome–DNA complexes considered. Importantly, this excellent match permits to information extraction not accessible in experiments, such as the liposome coverage Θ, opening routes to the exploration of adsorption isotherms in next contributions.

The main feature to highlight from the experimental data in Figure 9 is that the predicted distinct landmark of suspended particles holds. In particular, Figure 9 shows that Δ*D* ∼ |Δ*f*|^0.85^ which agrees with the predicted trends in Figure 8 (recall that |Δ*f*| ∝ Θ). Data from experiments with exosomes^25^ shown in the rightmost panel of Figure 9 further support this scaling. Notably, the sublinear scaling for the dissipation is even robust against the exosome size polydispersity and even when using more complex solvents, such as human serum. A similar scaling is observed for exosomes in pure HBS solvent (Figure 9 and Supporting Information of ref (25)). The best fit corresponds to Δ*D* ≈ 0.15|Δ*f*|^0.85^ for exosomes in human serum and Δ*D* ≈ 0.33|Δ*f*|^0.85^ for HBS solvent. In passing, a recent theoretical and numerical study predicts that the acoustic ratio decreases with the bending rigidity of liposomes,^16^ suggesting that exosomes become slightly more flexible when immersed in HBS solvent. In any case, note that neither the viscosity of the complex solvents used in ref (25) nor the length of the complex linker (which can be estimated to be about 10 nm) were reported in that study. The lack of this essential experimental data precludes a quantitative comparison with simulations. Yet, the QCM signals from exosomes present similar trends to that of liposome–DNA experiments and simulations, revealing the suspended configuration of these particles.

#### Adsorbed Particles

**Figure 10:**
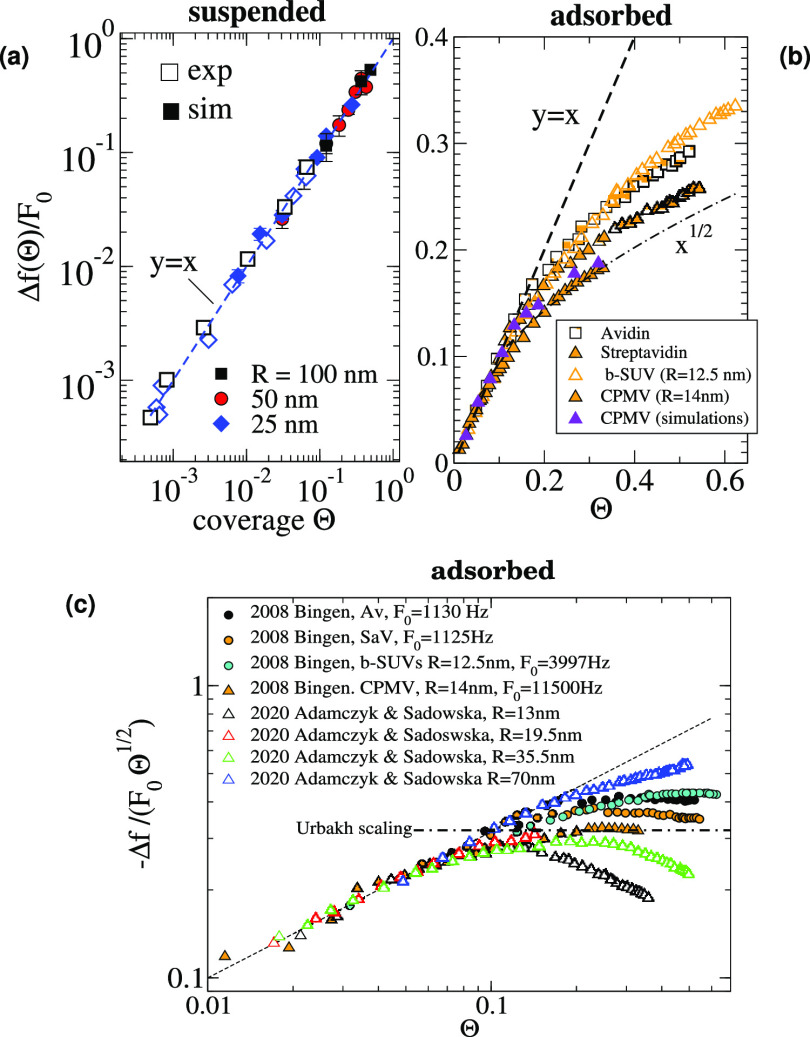
Frequency shift  scaled with the zero-coverage slope . Results for suspended (a) and adsorbed particles (b) are included for comparison. (a) Experimental data with liposomes suspended over DNA chains replotted from ref (26),^28^, and numerical results follow a linear scaling |Δ*f*| ∝ Θ. (b) In the case of adsorbates, deviations from the linear regime take place for Θ > 0.05 and roughly confirm the trend Δ*f* ∼ Θ^1/2^ predicted by Urbakh and Daikhin.^43^ (c) Frequency shift  scaled with Θ^1/2^ to compare coverage with the theoretical scaling derived by Urbakh and Daikhin.^43^ (b) Replots of the experimental data from Bingen et al. in ref (7) for avidin, streptavidin, virus capsids (CPMV), and biotin-SUVs (b-SUVs) and (c) data for adsorbed polymeric beads of radius 70, 35.5, 19, and 13 nm replotted from ref (8)

In order to compare with the available experiments on adsorbates, another series of simulations with an ensemble of (*N* = {1,10}), particles of radius *R* = 14 nm were placed under 25, 35, and 45 MHz, with coverages ranging from Θ = 0.027 to 0.27. The particles were placed touching the surface (Δ = 0), but no contact forces were added so as to reveal only the hydrodynamic effects. These simulations correspond to the experiments with CPMV published by Johannsmann et al.,^10^ which also included a numerical study in two dimensions. Numerical results for Δ*f* are in very good quantitative agreement with the experiments (see also Figure 10b). However, although the essential features for Δ*D* were found to be similar to experimental results (e.g., a nonmonotonous trend against the coverage), some deviations were found. The present 3D simulations predict the maximum value of Δ*D* to be at Θ ≈ 0.1 (while for *R* = 50 nm, it was located around Θ ≈ 0.2, see the inset of Figure 7). Experiments with viruses show this maximum at Θ ≈ 0.15.^10^ It is noted that 2D simulations of rigidly adsorbed particles presented in the original contribution^10^ predict this maximum to be at larger coverage Θ ≈ 0.25. Although 3D results are closer to experimental results, it seems that the contact forces (and probably the virus viscoelasticity) are essential to unveil these fine-tuned details.

Figure 10 collects results for Δ*f* from a series of experiments involving different types of adsorbates. The figure also includes simulation results for a collection of *R* = 14 nm particles (at Δ = 0) representing the conditions of the experiments with CPMVs.^10,30^ To focus on scaling trends, the frequency shift presented in scaled form, , where the frequency-per-coverage at infinite dilution, , is a well-defined limit as |Δ*f*| scales linearly with Θ for low enough coverage. Figure 10 compares the suspended and adsorbed configurations to clearly illustrate the qualitatively different behaviors. While the linear scaling |Δ*f*| ∝ Θ remains valid in suspended particles up to large coverage, in the case of adsorbates, the linear scaling is already lost approximately for Θ > 0.05. At higher coverages, the behavior of |Δ*f*| is not universal and highly depends on the analyte–substrate–solvent combination, reflecting relevant effects from contact forces, analyte distributions, and possibly the viscoelastic response of the adsorbates. In the high coverage range, it is reasonable to compare the experimental results with the purely hydrodynamic theory by Urbakh et al.^43^ for corrugated surfaces with a given height and rugosity as it corresponds to a picture which is consistent with an ensemble of small and rigidly attached adsorbates. Indeed, this trend is consistent with some of the experimental results, particularly those for *R* = 14 nm viruses.^10,30^ However, for Θ > 0.05, trends for different analytes do not collapse to a master curve. In order to more closely inspect the behavior of Δ*f* beyond the dilute regime, Figure 10c presents additional experimental data scaled as , which should reach a constant at high coverage, provided the validity of the Urbakh scaling law |Δ*f*| ∼ Θ^1/2^. Clearly, significant deviations from the Θ^1/2^ scaling are observed, which cannot be easily rationalized in terms of the particle size. Instead, it is reasonable to conclude that specific particle–substrate interactions and molecular mechanical properties induce measurable effects and different trends at high enough coverage (precisely, such a large sensitivity is the interesting feature of QCM). While it cannot be taken as a general trend, Figure 10c suggests that small adsorbates tend to fulfill the corrugated surface scaling Θ^1/2^, while larger particles exhibit different scalings (e.g., Δ*f* ∼ Θ^0.75^ for 70 nm radius polymeric particles^8^) which are closer to the suspended behavior Δ*f* ∼ Θ. This fact suggests a larger influence of the hydrodynamics created by mass distributed further away from the resonator.

The following discussion presents the implications of the present findings on standard procedures used in applied QCM analyses. Then, it comments on the theory by Leshansky et al.^17^ concerning the impedance of the adsorbed state and the suspended-to-adsorbed transition.

### Implications for Applied QCM

#### Acoustic Ratio at Low Coverage

**Figure 11:**
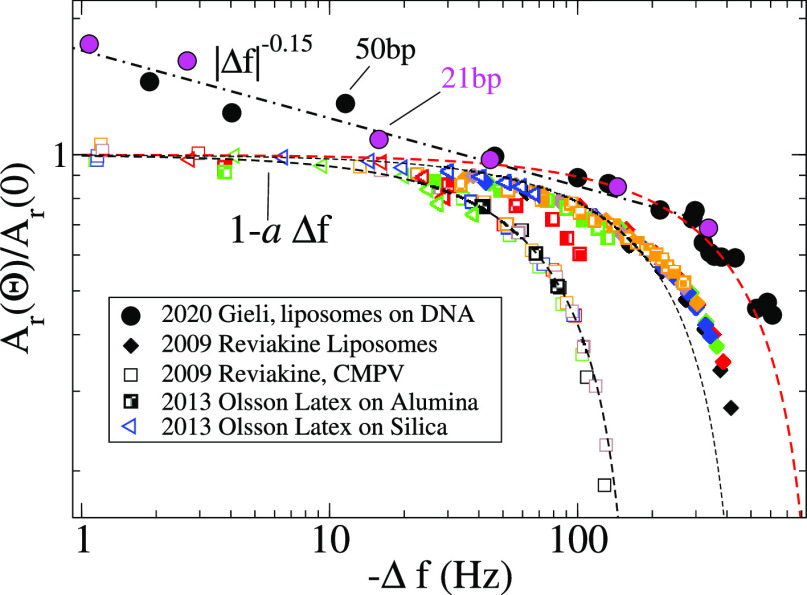
Experimental values of the acoustic ratio A_r_(Θ) = *f*_n_Δ*D*/|Δ*f*| normalized with the zero-coverage intercept A_r_(0) (see text). Filled circles correspond to the experimental data from ref (26) for suspended liposomes tethered to DNA strands of 21 and 50 bp length. Hatched symbols correspond to data from experiments with adsorbed particles: liposomes,^11,30^ viruses,^11^ and latex nanoparticles on silica or on alumina-coated surfaces.^5^ The dotted–dashed line indicates the power law |Δ*f*|^–0.15^, and dashed lines indicate the linear relation 1 – *a*|Δ*f*|, which fits relatively well with data for adsorbed analytes at low frequency.

Due to its relevance in QCM analyses, it is interesting to consider the implications of the present findings on the acoustic ratio A_r_ ≡ −*f*_n_Δ*D*/Δ*f*, starting with analyzing the small coverage limit (i.e., small |Δ*f*|). As shown in Figure 10b, experiments with deposited analytes (particles in the range *R* < *O*(100)nm) present Δ*f* ∼ Θ at low coverage and nonmonotonous Δ*D*(Θ) dependence. The acoustic ratio thus decays when plotted in a A_r_ – |Δ*f*| chart,^30^ and for a not-so-large |Δ*f*|, it can be fitted with a linear law A_r_(Θ) = A_r_(0) – *a* |Δ*f*|, where *a* and A_r_(0) are case-dependent constants. The intercept A_r_(0) (which has been sometimes called “dissipation capacity”^14,26^) is often used to compare samples at infinite dilution (Δ*f* → 0), where hydrodynamic couplings between the analytes are expected to be irrelevant. The present findings indicate, however, that in the case of suspended particles, the low-coverage acoustic ratio should scale as A_r_ ∼ Θ^–0.15^ (as 1 – β ≈ – 0.15), implying that it is not accurate to use A_r_(0) to characterize the sample. This fact is illustrated with the experimental results in Figure 11, where a systematic drift A_r_ ∼ |Δ*f*|^–0.15^ is revealed at low values of |Δ*f*|. On the contrary, the acoustic ratio of adsorbed particles presents a well-defined (or more properly, experimentally reachable) Δ*f* → 0 limit.

#### Δ*D* = 0 Extrapolation Method for Particle Size Estimation

Another popular procedure involving the A_r_ – |Δ*f*| chart is based on the large coverage limit and the decrease of Δ*D* with Θ. The extrapolation method used in many QCM analyses consist determination of the frequency shift for which A_r_(|Δ*f*|) crosses zero (|Δ*f*| = A_r_(0)/*a* for the linear fit). As Δ*D* = 0 has been associated with a rigid film behavior, this phenomenon has been ascribed to the formation of a closely packed rigid layer of adsorbates. In such a situation, the hydrodynamic details should also be irrelevant, allowing the use of the SR^3,9^ to estimate the adsorbed mass. Then, knowing the particle density and assuming close packing Θ ≃ 0.57, the adsorbed mass density leads to the particle size. Although useful in some cases,^5,11,13^ the extrapolation method is not always reliable^9,16,44^ (see also the comments in ref (11) about strong deviations for higher harmonics). The present results show that the decrease of Δ*D* (with Θ and for Δ ≈ 0) is due to the perturbative flow structure, and it might take place below close packing (see also ref (16)). For instance, extrapolating the results in Figure 3 (Θ = 0.49) suggests a crossover to Δ*D* = 0 around Δ ∼ 0.01*R*. As shown in Figure 7 at high enough coverage, Δ*D* (or −Re[*Z*]) decreases abruptly as the particle approaches the surface (a feature which is also observed in suspended plates^16^). In conclusion, the loci of the Δ*D* = 0 crossover in the Δ – Θ chart are certainly not evident, and numerically, it would require using extremely fine meshing.

#### Adsorption Isothermal and Dissociation Constant Determinations from Δ*f*

Many QCM studies on molecular binding and on the evaluation of dissociation constants *K*_D_ construct the Langmuir adsorption isothermal Θ/Θ_max_ = *c*/(*c* + *K*_D_) (bulk molar concentration *c*) using |Δ*f*| as proxy to the analyte coverage Θ. Yet, Figure 10 shows that if the particles are adsorbed, this procedure may lead to substantial errors (particularly, if *K*_D_ is not large enough to be able to approximate |Δ*f*|/|Δ*f*|_max_ ≈ *c*/*K*_D_ in the linear regime, i.e., Δ*f* ∝ Θ for Θ < 0.05). In line with this, the present findings provide a positive result and open a route for |Δ*f*|-based adsorption isotherms in the case of suspended analytes (typically, receptor–ligand systems) for which |Δ*f*| ∝ Θ up to high coverage.

### Suspended–Adsorbed Transition: Comments on the Theory by Leshansky et al.

**Figure 12:**
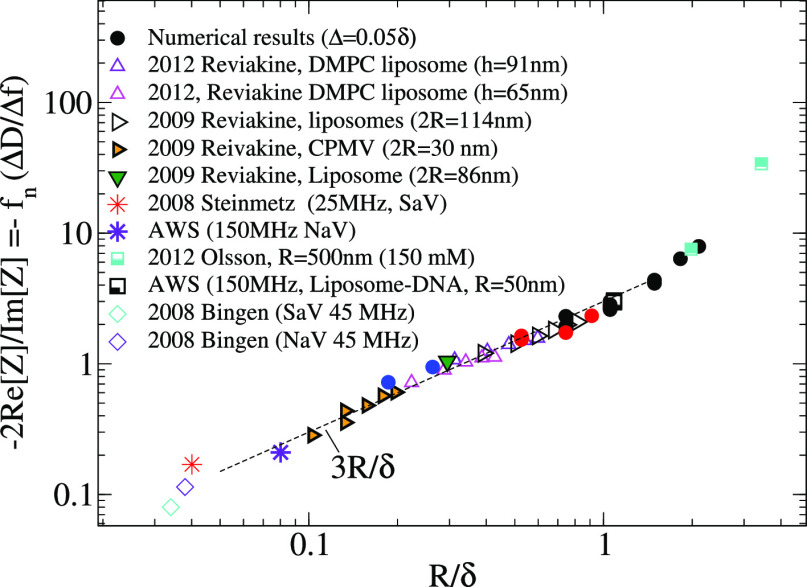
Experimental values of the acoustic ratio obtained in several systems (*A*_r_ = −lim_Δ*f*→0_*f*(Δ*D*/Δ*f*)) along with numerical calculations (filled circles) for “nearly touching” spheres Δ ≃ 0.05δ at small coverage. The experimental works are cited in the main text. Data replotted for 2012 Olsson^6^ corresponds to *R* = 500 nm silica particles in a silica substrate immersed in a 150 mM KCl buffer to ensure strong adhesion.

Results reported by Leshansky’s et al.^17^ for the impedance of the adsorbed state lead to strong deviations from the experimental results. For instance, in the range *R*/δ < 2, the adsorbed impedance leads to Δ*f* > 0 and Δ*D* < 0, while experimental evidence indicates Δ*f* < 0 and Δ*D* > 0. This problem is relevant because it provides an essential piece of information to understand the transition from suspended (but nearly touching Δ ≈ 0) to the adsorbed state (Δ = 0, where the contact forces are generally present). Experimental evidence suggests that the suspended-to-adsorbed transition is not abrupt (otherwise, QCM signals would present strong jumps upon particle adsorption events). This observation is at odds with the conclusion of ref (17). Moreover, numerical calculations with “nearly touching” particles are found to be consistent with experimental trends [e.g., Figure 10b]. More evidence is presented in Figure 12 by comparing the acoustic ratio (at vanishing coverage limit A_r_ = −lim_Δ*f*→0_*f*(Δ*D*/Δ*f*)) obtained from experiments with disparate adsorbates (in size and type) and numerical results for Δ ∼ 0.05δ.

This section retakes this problem in the framework of the theory^17^ by adding the contribution of the contact forces. In what follows, the same scaling units are used as in ref (17) (length, velocity, force, and impedance units are *R*, *v*_wall_ and η*Rv*_wall_, and η*nR*). In these units, the wall force equals the impedance *F* = *Z*). The elegant approach derived in ref (17), based on the LRT, starts by evaluating the excess impedance (or equivalently the excess wall force) due to a stationary particle over a moving surface, *F*_s_. Recall that excess refers to the difference with respect to the base flow contribution, whose velocity is **v**_0_ = *v*_0_(*z*)**x** with *v*_0_(*z*) = *e*^–α*z*^. Using the perturbative flow **ṽ** as dual flow for the LRT, they derive , where σ^[1,0]^ is the stress created by a stationary sphere under a moving wall (**v**(*z* = 0) = **x** and **v**(*r* = 1) = 0) and  corresponds to the perturbation of a stationary particle on the Stokes flow, with boundary conditions **ṽ**(*z* = 0) = 0 and . In an attempt to simplify the notation, the superscript will denote the boundary conditions at wall and particle: i.e., [**v**(*z* = 0), **v**(*r* = 1)]. The above relation clearly reflects the transmission of stress from the suspended particle to the QCM surface. Invoking linear superposition of the flows, the impedance of a particle translating with *x*-velocity (*V* = *u*_*x*_) and rotating with *y*-angular velocity (Ω = Ω_*y*_) over the oscillatory *z* = 0 surface can be written as *F* = *F*_s_ + *AV* + *B*Ω where the resistances *A* and *B* are, respectively, the plane tractions created by particles with velocities (*V*, Ω) = (1, 0) and (0,1). Using the LRT, one finds *A* = ∫_*z*=0_σ_*xz*_^[0,1]^d*x*d*y* = ∮_*r*=1_σ_*xz*_^[1,0]^d*r*^2^, and the corresponding relation for the rotational resistance *B*. In the adsorbed state (*V*, Ω) = (1, 0), the adsorbates’ impedance deduced in ref (17) is then *F*_a_ = *F*_s_ + *A*. On reaching this conclusion, the contact force and torque were neglected. Yet, unless some other external device (e.g., a putative optical tweezer) is used, the force needed to ensure the adsorbed condition (*V* = 1 and Ω = 0) has to be exerted by the QCM wall, and this force needs to be added to the impedance (see the total wall stress in eq 5). Adding the contact force – **F**_c_ and torque – τ_c_ exerted by the wall to the particle (thus the minus sign), the translational and rotational particle equations of motion are ξα^2^*V* = *F*_drag_ – *F*_c_ (in *x*-direction) and (2/5)ξα^2^Ω = τ_drag_ – τ_c_ (in *y*-direction). As in ref (17), ξ ≡ (4π/3)ρ_p_/ρ, where ρ_p_ is the particle density, and the factor two-fifths comes from the momentum of inertia of a sphere. The drag force (traction on the sphere) is *F*_drag_ = ∮_*r*=1_σ_*xr*_d*r*^2^ (here, σ is the stress in the real QCM setup, i.e., a moving particle under the oscillatory surface). In the rigidly adsorbed state, a strong enough particle–substrate interaction ensures that *V* = 1 and Ω = 0 so that *F*_c_ = *F*_drag_ – ξα^2^ and τ_drag_ = τ_c_.

To express these relations in terms of the theoretical framework of ref (17), one needs to follow them using another clever idea, which avoids explicitly integrating the particle dynamics. Again, the LRT permits relating *V* and Ω for the QCM flow (**v**(*z* = 0) = **x**) with the (symmetric) resistance matrix  of a particle translating and rotating over a stationary wall (**v**(*z* = 0) = 0) with velocities .^17^ These  can be then set to (1,0) and (0,1) to write the particle equations of motion
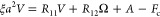
7
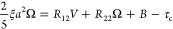
8where I have included additional terms here for the contact force and torque. Therefore, for a rigidly adsorbed particle (*V*, Ω) = (1, 0)
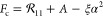
9leading to a “contact” impedance
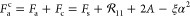
10

Note that for (*V*, Ω) = (1, 0), we have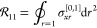 and
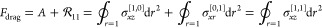
Using the LRT, *F*_c_ = ∫_*z*=0_σ_*xz*_^[0,1]^d*x*d*y* + ∮_*r*=1_σ_*xr*_^[0,1]^d*r*^2^ – ξα^2^, which sums up the particle inertia and the tractions on the sphere and surface due to a sphere moving under a stationary wall. The additional “hydrodynamic” force on the surface comes from the perturbative flow in the QCM setting:  (moving particle under the moving wall), which has opposite sign to *F*_a_^c^. While this approach to *F*_c_ corresponds to the rigidly adsorbed limit (*V*, Ω) = (1, 0), this delicate part of the problem opens important (molecular-specific) questions in the QCM field. In general, the contact force (and torque) may not be strong enough to ensure (*V*, Ω) = (1, 0). In this case, one needs to solve eqs 7 and 8, and the impedance will be modified (both because of the resulting hydrodynamic perturbation due to the particle motion and to the possibly viscoelastic particle–substrate response).

In the absence of lubrication forces (Δ = 0), the contact force *F*_c_ (and torque) ensures (*V*, Ω) = (1, 0) in the rigidly attached case. If the particle is not touching the surface (no contact forces are present), lubrication forces lead to *V* → 1 and Ω → 0 as Δ → 0 and probably divergent resistances *A* and *B* (the impedance being *F* = *F*_a_ + *A*(*V* – 1) + *B*Ω). While it is reasonable to say that as Δ > 0 is decreased, lubrication forces become comparable to the contact forces needed to rigidly attach the particle; strictly at Δ = 0, the lubrication forces cease to exist, so that the limit is conceptually delicate. Taking results for *F* and *F*_a_ at Δ ∼ 0.1*R* and *R*/δ = 0.25 from ref (17) (and using ), a gross estimation yields Re[*Z*_a_^c^] ≈ −1. Also, taking the analytical relations for the long-distance approximation (shown to be fairly good for small *R*/δ and up to Δ/*R* ∼ 0.1^17^) one gets, at contact, Re[*Z*_a_^c^] ≈ −0.6 and Im[*Z*_a_^c^] ≈ 3.6. These estimations are consistent with the experimental results (i.e., Im[*Z*_a_^c^] > 0 and Re[*Z*_a_^c^] < 0) and not far from the present results for Z close to the wall and from the numerical results for free particles at Δ/*R* ≈ 0.1 [Figure 6 (bottom)] reported by Leshansky et al. While this suggests a nonabrupt suspended-to-adsorbed transition (i.e., not one with *Z* changing sign), the impedance may present significant variations near the wall (see, e.g., Figure 7 for Re[*Z*]). In passing, this effect could be the origin of the overshoots in Δ*D* experimentally observed in the transient regime of adsorption.^10^ This interesting problem is thus still open. While it might be experimentally difficult to ideally attain the “rigid-sphere” Δ → 0 limit due to surface rugosity and molecular viscoelasticity effects, more accurate theoretical analyses are necessary to frame finer details. This will require tracking the strong lubrication forces and derive scalings for very small Δ. The Δ → 0 limit in ref (17) leads to *Z* ≈ *Z*_a_ – (4π/5)(4*C*_1_ – *C*_2_)e^–αR^, where (*R*/δ-dependent) *C*_1_ and *C*_2_ determine the particle velocities [*V* – 1 = −*C*_1_/ln(Δ/*R*) + *O*(1) and Ω = −*C*_2_/ln(Δ/*R*) + *O*(1)]. To theoretically determine *C*_*i*_ and *O*(1) contributions, the problem resides in matching the lubricating layer limit solution with the outer flow, where the wall reaction flow might be still relevant. Numerically, a fluid solver based on standard boundary conditions (over the full domain) will become computationally impractical (as it requires tall boxes and small meshing *h*/δ and *h*/*R*). Alternative schemes with open flow in doubly periodic systems, in the spirit of ref (45), are being now considered in our group. Future work in this direction is being planned.

## Conclusions

To summarize, numerical calculations based on the IB method and experimental results for quite different analytes reveal qualitatively different trends for the coverage scaling of QCM signals in the case of suspended Δ > 0 and adsorbed Δ = 0 particles. In the suspended configuration, Δ*f* ∝ Θ for the whole range of coverages (0 < Θ < 0.5), while Δ*D* ∼ Θ^β^ [for Θ_l_ < Θ < 0.5, where Θ_l_ ∼ *O*(0.01) is some, yet to be determined, ultra-low-coverage crossover from linear to sublinear scaling]. For particle radius 0.25 < *R*/δ < 1, the exponent is found to be β ≈ 0.85 (it slightly decreases for smaller particles). These general scaling laws are not to be found in the case of adsorbates. Experimental trends show that Δ*f* ∼ Θ for Θ < 0.05, but they present quite different scalings at larger coverage depending on the combination of the substrate, solvent, and particle type. The dissipation of adsorbates reaches a maximum around Θ ∼ 0.2 and then decreases at high coverage; this feature is a long-standing question in the QCM field.^3,10^ Simulations indicate that this phenomenon is due to the modifications in the vortical structure of the hydrodynamic perturbation at ω*t* = 0, taking place (even without subtract-particle forces) at a small enough gap distance (Δ ≈ 0) and large enough Θ.

This work and previous contributions^9,16,17^ clearly show that hydrodynamics is a dominant source of impedance. However, there are other relevant effects that modify the QCM response. Notably, the interfaces create singular contributions such as fluid slippage under particular wetting conditions (e.g., strong hydrophobicity), which tend to reduce the transmission of tangential stress to the resonator and thus the impedance. In fact, QCM has been recently used to unveil slippage in moving fluids,^46^ with consequences in biochemical sensors^47^ and possibly modifying the relation between Δ*f* and the coverage (making it linear) of quasi-adsorbed spheres and spheroids.^48^ If the particles are strongly hydrophobic, fluid slippage on the particle–fluid interface might also alter the hydrodynamic transmission of momentum to the resonator even if the particle is suspended, an effect which has not been so far studied. Moreover, elastic and viscoelastic responses of soft (deformable) analytes and soft substrate layers (e.g., membranes) might lead to a variety of phenomena even in suspended configurations.^49^ Nonlinear lift forces have also been predicted in QCM in rigid particles and no-slip surfaces.^50^ Concerning the dynamic picture, note that each QCM measurement samples a quasi-instantaneous “frozen” snapshot of the Brownian motion of colloids. Albeit, a particle of radius *R* = 10 nm will displace by diffusion its own radius over 100 cycles at *f* = 10 MHz possibly leading to, yet unstudied, memory effects in ring-down QCM-D signals of tethered proteins. Adsorbed particles might experience quite different physicochemical forces,^51^ including dispersion (van der Waals) forces, electrostatic forces, hydrophobic forces, and even fluctuation forces in the case of soft objects (e.g., vesicles). These molecular forces occur at few nanometer distance, and in this sense, QCM is an inherent multiscale problem. Mesoscopic approaches, such as the one presented in this work, should be gradually able to deal with these molecular details via coarse-grained methodologies^52^ designed to formally connect the molecular complexity with mesoscopic (generally *viscoelastic*) forces. This effort should be guided by experiments, and it demands multidisciplinary collaborations with experts from disparate open communities.
